# A rapid and cost-effective Chelex-100 resin-based method for genomic DNA extraction from adult zebrafish tissues

**DOI:** 10.1371/journal.pone.0358591

**Published:** 2026-09-18

**Authors:** Wei Feng, Boya Li, Yiqiong Niu, Qiannan Xu, Xiandun Zhai

**Affiliations:** 1 School of Basic Medicine and Forensic Medicine, Henan University of Science and Technology, Luoyang, China; 2 Institute of Special Medicine, Henan University of Science and Technology, Luoyang, China; 3 Forensic Center, Henan University of Science and Technology, Luoyang, China; University of the Faroe Islands: Frodskaparsetur Foroya, FAROE ISLANDS

## Abstract

The zebrafish (*Danio rerio*) is a widely used vertebrate model in biomedical research, yet the rapid and cost-effective extraction of genomic DNA (gDNA) from adult zebrafish tissue remains a practical challenge for routine molecular applications. To address this, we systematically optimized and evaluated a Chelex-100/proteinase K-based extraction protocol across eight adult zebrafish tissues (muscle, liver, heart, spleen, kidney, intestine, brain, and eggs) and compared its performance against that of a commercial kit. The optimized protocol, involving incubation at 56 °C for 15 min and boiling at 100 °C for 10 min, required approximately 25 min per sample. Across all tested tissues (3 biological replicates per tissue), the Chelex-100 method yielded gDNA concentrations ranging from 177.2 ± 3.5 ng/µL to 674.5 ± 22.1 ng/µL per 5 mg of tissue, which were generally higher than those obtained with the commercial kit. However, the purity (A260/A280 ratios) was consistently lower for the Chelex-100 method (1.36–1.83) than for the kit (1.84–2.02), indicating the coextraction of proteinaceous impurities. Despite this, Chelex-extracted gDNA supported successful PCR amplification and Sanger sequencing of target fragments, with consistent amplicon quality. In real-time PCR assays, both methods produced amplification curves, but the Chelex-100 method yielded systematically higher Ct values, suggesting reduced sensitivity with less-pure templates. Crucially, the Chelex-100 method avoided hazardous organic solvents and required no column purification, with an estimated reagent cost of <$0.20 per sample versus approximately $2.00 per sample for the commercial kit. We conclude that this optimized Chelex-100/proteinase K protocol provides a rapid, economical, and operationally simple alternative for routine gDNA extraction from adult zebrafish tissues and is particularly suitable for PCR-based genotyping, sequencing and qPCR applications, though its lower purity warrants careful consideration for high-sensitivity quantitative assays.

## 1. Introduction

The zebrafish (*Danio rerio*) has emerged as one of the most versatile model organisms because of its high fecundity, the transparency of its early embryos, its rapid embryonic development, its low maintenance costs, and its high genetic and physiological similarity to humans, sharing approximately 70% gene homology and 82% conservation of human disease-associated genes [1–8]. These traits make the zebrafish particularly well suited for modeling various human diseases, including cardiovascular health, neurological diseases, and metabolic dysfunctions, as well as for toxicology assessments and high-throughput drug screening [9–14]. Therefore, reliable genomic DNA (gDNA) extraction from zebrafish tissues is a fundamental requirement for genotyping, sequencing, and gene function studies. However, current methods for obtaining gDNA from adult zebrafish tissues, such as phenol–chloroform extraction and commercial kit-based methods, provide high purity but are often time-consuming, labor-intensive, costly, or require hazardous reagents, which limits their suitability for routine, rapid, and high-throughput applications [15–20]. These limitations create a need for a simpler and more economical DNA extraction method that can reliably support downstream molecular analyses.

Chelex-100 is a chelating resin that binds divalent metal ions to inactivate nucleases, removes PCR inhibitors, and disrupts cells under alkaline and boiling conditions, thereby helping to release DNA from cells and protect DNA from nuclease-mediated degradation [21–23]. Chelex-based DNA extraction has been widely used for forensic trace samples, microorganisms, insects, rodents, seafood, and plants, supporting applications in forensic identification, sequencing, genotyping, pathogen detection, and other areas owing to its operational simplicity, low risk of contamination, and minimal sample loss [24–34]. However, there are few studies on its application to adult zebrafish tissues, particularly with respect to obtaining DNA of adequate quality for direct PCR analysis from multiple tissue types. To address this gap, we developed a rapid and straightforward Chelex-100/proteinase K-based protocol for gDNA extraction from adult zebrafish tissues that reliably supports downstream PCR detection without the need for organic solvents or column-based purification. This method minimizes the number of handling steps and reduces time and cost, providing a practical alternative to conventional extraction methods for routine molecular research on zebrafish in biomedicine, environmental science, pharmacology, agriculture, and fisheries.

## 2. Materials and methods

### 2.1. Experimental ﬁsh

Approximately six-month-old wild-type AB zebrafish were purchased from Shandong YiXiYue Biotechnology Co., Ltd., and maintained in a circulating water system following standard guidelines [35]. The zebrafish were housed at 28 ± 0.5 °C with a 14 h/10 h light/dark cycle and a pH of 7.2–7.4, and they were fed zebrafish-specific feed twice a day. Only healthy fish, whose mean body length and weight were 3.5 ± 0.2 cm and 0.5 ± 0.05 g, respectively, were used in the experiments. All experimental procedures were performed in accordance with the ethical guidelines approved by the Institutional Animal Care and Use Committee of Henan University of Science and Technology (ethical approval no. HAUST-025-F0306016). This study was designed and reported following the ARRIVE guidelines.

### 2.2. Sample collection

Adult zebrafish (male: female = 1:1) were anesthetized with tricaine methanesulfonate (MS-222), and various tissues (muscle, liver, heart, spleen, kidney, intestine, brain, and eggs) were dissected. For each tissue type, samples from one male and one female fish were pooled into a single microfuge tube to serve as an independent biological replicate (n = 3 biological replicates per tissue type) and standardized to 3–5 mg using a precision balance to minimize variation arising from sample weight. Each sample was then either processed immediately for DNA extraction or stored at –80 °C until use.

### 2.3. Nucleic acid extraction

#### 2.3.1. Optimization of gDNA extraction using Chelex‑100 resin.

5 mg of zebrafish muscle tissue was placed in a microfuge tube containing 300 μL of 5% Chelex‑100 resin (Bio‑Rad, Hercules, CA, USA) and 2 μL of proteinase K (20 mg/mL; 9034, Takara, Japan). The mixture was vortexed for 10 s and spun at 13,680 × g for 3 min. The samples were incubated at 56 °C for different durations (5, 15, 30, and 60 min), vortexed for 10 s, and boiled at 100 °C for 8 min. After boiling, the samples were vortexed for 30 s and spun at 13,680 × g for 3 min. The purity and concentration of the supernatants were determined by measuring the A_260_/A_280_ ratio using a NanoDrop 2000 spectrophotometer (Thermo Fisher Scientific, Waltham, MA, USA). The optimal incubation time was defined as the duration at which the highest DNA concentration was obtained. Based on the initial results, the boiling time was further optimized (5, 8, or 10 min) using the optimal incubation time (15 min) determined in the first step. Each optimization condition was tested with three independent biological replicates.

#### 2.3.2. Chelex-100 resin-based gDNA extraction from other zebrafish tissues.

Zebrafish tissues (liver, heart, spleen, kidney, intestine, brain, or eggs) were supplemented with 5% Chelex-100 resin and proteinase K at ratios of 60 μL and 0.4 μL per 1 mg of tissue, respectively. The mixture was then vortexed for 10 s and centrifuged at 13,680 × g for 3 min. The samples were heated at 56 °C for 15 min, vortexed for 10 s, and denatured at 100 °C for 10 min. Subsequently, the samples were vortexed for 30 s and centrifuged at 13,680 × g for 3 min. Approximately 40 μL of the supernatant per 1 mg of tissue was transferred to a new tube for immediate use or storage. The purity and concentration of the extracted DNA were assessed as described above. The extraction experiment for each tissue type was repeated three times.

#### 2.3.3. Commercial kit-based gDNA extraction.

gDNA was extracted from zebrafish tissues using a tissue DNA kit (D3396, OMEGA Bio-Tek, Norcross, GA, USA) according to the manufacturer’s instructions. Briefly, 3–5 mg of tissue was lysed in 200 μL of TL buffer and 25 μL of OB protease at 55 °C for 3 h in a shaking water bath. After centrifugation, the supernatant was transferred to a new tube, mixed with 220 μL of BL buffer, and incubated at 70 °C for 10 min. Following the addition of 220 μL of 100% ethanol, the lysate was applied to a HiBind® DNA Mini Column, washed with HBC and DNA Wash Buffers, and centrifuged. DNA was eluted with preheated elution buffer to a final volume of approximately 85 μL. Purity and concentration were evaluated as described above. All experiments were conducted with three independent biological replicates.

### 2.4. Quality assessment by PCR, DNA sequencing and real-time PCR

gDNA extracted by both methods was used as template for PCR, DNA sequencing and real-time PCR (qPCR). The primers of β-actin, 18S rRNA, TLR9 and ikbaA were synthesized by Shanghai Generay Biotechnology Engineering Co. Ltd. and the sequences are shown in S1 Table. The 20 μL PCR reaction volume contained 10 μL of *Premix Taq* (RR902A; Takara, Japan), 0.8 μL each of 10 μM forward and reverse primers, 2 μL of diluted template DNA, and 6.4 μL of sterile water. Amplification was performed in an Applied Biosystems 9700 PCR System (Applied Biosystems, Foster City, CA, USA) under the following conditions: initial denaturation at 95 °C for 3 min, followed by 35 cycles of 95 °C for 30 s, 58 °C–70 °C for 30 s, and 72 °C for 1 min, with a ﬁnal extension at 72 °C for 8 min. The resulting amplicons were analyzed by 2% agarose gel electrophoresis and the amplicons of the TLR9 gene were sequenced at Sangon Biotech (Shanghai) Co., Ltd. qPCR was performed in a total volume of 20 μL containing 10 μL of TB Green *Premix Ex Taq* II (RR820A, Takara, Japan), 0.8 μL each of 10 μM forward and reverse primers, 1.0 μL diluted template DNA and 7.4 μL of sterile water. The amplification conditions for the qPCR were as follows: an initial denaturation step at 95 °C for 30 s, followed by 40 cycles of 95 °C for 5 s, 58 °C for 30 s and 72 °C for 30 s. In all cases, each qPCR trial was performed in triplicate and repeated at least three times.

### 2.5. Statistical analysis

All statistical analyses were performed using GraphPad Prism 10.1.2 (GraphPad Software, CA, USA). The data are presented as the mean ± standard deviation (SD). Pairwise comparisons of the extracted gDNA concentration from muscle using the Chelex‑100 resin method at different incubation or boiling times were evaluated by the one‑way analysis of variance followed by Tukey’s test, and the gDNA concentrations across various tissues using two different methods were evaluated by the two-tailed T-test. A *p*‑value < 0.05 was considered statistically significant. The experiments involving gDNA extraction using the Chelex-100 resin method or the commercial kit were performed independently with at least three biological replicates.

## 3. Results

### 3.1. Optimization of Chelex-100 resin-based gDNA extraction from zebrafish muscle

**Fig 1:**
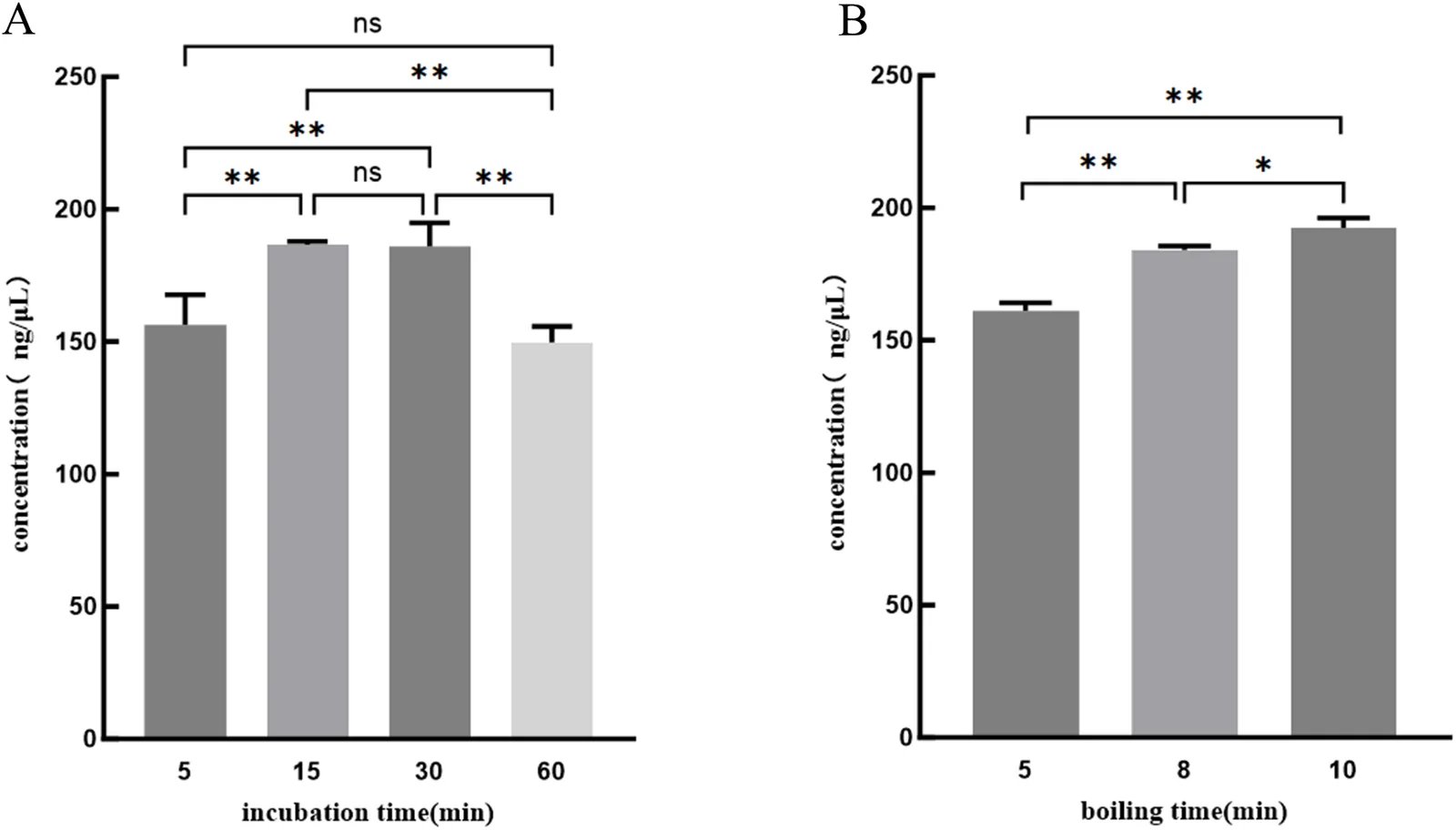
Optimization of gDNA extraction from zebrafish muscle using the Chelex‑100 resin method. (A) DNA concentration obtained at 56 °C for different incubation times (5, 15, 30 and 60 min) was measured using a NanoDrop 2000 spectrophotometer. (B) DNA concentration obtained at 100 °C for different boiling times (5, 8 and 10 min) following the optimized incubation condition described above. *, *P* < 0.05; **, *P* < 0.01; ns, not significant.

To optimize the Chelex-100 resin method, gDNA was extracted from zebrafish muscle using different incubation times (5, 15, 30, and 60 min) at 56 °C, and the concentration and purity (A260/A280 ratio) were measured. The resulting DNA concentrations were 156.4 ± 11.2, 186.6 ± 1.2, 186.0 ± 8.8, and 149.7 ± 6.1 ng/μL, respectively, with corresponding purity values of 1.33 ± 0.02, 1.28 ± 0.01, 1.29 ± 0.01, and 1.28 ± 0.01, respectively (Fig 1A). The concentration data revealed a sharp increase from 5 min to a peak at 15 min, remained effectively plateaued at 30 min, and then drastically decreased to the lowest value at 60 min. The A260/A280 purity ratios remained remarkably consistent across all four incubation durations. Moreover, the concentrations did not significantly differ between 15 and 30 min of incubation (Fig 1A, *P* = 0.99). Thus, we selected 15 min of incubation for the subsequent optimization steps. Different boiling times (5, 8, and 10 min) at 100 °C were subsequently evaluated under the established incubation conditions. The resulting DNA concentrations were 161.1 ± 3.0, 184.1 ± 1.5, and 192.4 ± 3.7 ng/μL, with purity values of 1.32 ± 0.004, 1.34 ± 0.01, and 1.37 ± 0.01, respectively. The data revealed that boiling for 10 min resulted in the highest DNA concentration, with statistically significant differences among the groups (Fig 1B, *P* < 0.01 or *P* < 0.05). Therefore, a boiling time of 10 min was determined to be optimal. Based on these findings, the optimized protocol for gDNA extraction from zebrafish muscle using Chelex-100 resin and proteinase K consists of incubation at 56 °C for 15 min, followed by boiling at 100 °C for 10 min.

### 3.2. gDNA extraction from various zebrafish tissues using the Chelex-100 resin method and a commercial kit

gDNA was successfully extracted from multiple zebrafish tissues, including muscle, liver, heart, spleen, kidney, intestine, brain, and eggs, using either the Chelex-100 resin method or a commercial DNA extraction kit. The DNA concentration and purity per 5 mg of starting material were compared between the two methods and are shown in Table 1. The DNA concentrations obtained using the Chelex-100 resin method were generally significantly greater than those obtained using the commercial kit (*p* < 0.01), except in the liver and spleen tissues. The A260/A280 absorbance ratio, which ranged from 1.36 to 1.83 when the Chelex-100 resin method was used, was lower than that obtained with the commercial kit (1.84 to 2.02), indicating that the lower purity of the extracted gDNA was likely attributable to co-extraction of contaminants resulting from the absence of purification steps. As shown in Table 2, comparative analysis revealed that the Chelex-100 resin method had advantages in terms of the time needed, the avoidance of toxic compounds, and the cost estimate per sample (in USD), but disadvantages in terms of purity.

**Table 1:** Concentration and purity of gDNA extracted from different adult zebrafish tissues using the Chelex‑100 resin method and a commercial kit.

Tissue (5 mg)	Chelex-100 method		Commercial kit		*P* value
	Concentration (ng/µL)	A260/A280	Concentration (ng/µL)	A260/A280	
muscle	177.2 ± 3.5	1.36 ± 0.01	33.7 ± 2.0	1.84 ± 0.03	<0.0001
liver	631.3 ± 6.6	1.83 ± 0.05	636.0 ± 5.2	1.88 ± 0.08	0.2178
eggs	519.4 ± 17.2	1.61 ± 0.08	335.9 ± 12.2	2.01 ± 0.08	0.0080
spleen	674.5 ± 22.1	1.74 ± 0.01	671.4 ± 19.7	1.97 ± 0.06	0.9089
heart	275.0 ± 4.32	1.62 ± 0.02	215.6 ± 9.9	1.95 ± 0.06	0.0075
kidney	461.4 ± 12.2	1.57 ± 0.04	354.4 ± 2.6	1.99 ± 0.07	0.0060
intestine	568.5 ± 8.8	1.65 ± 0.03	348.7 ± 10.8	2.02 ± 0.05	0.0008
brain	236.6 ± 11.2	1.61 ± 0.04	182.5 ± 9.1	1.99 ± 0.04	0.0038

**Table 2:** Comparison of the two gDNA extraction methods.

Extraction Method	Time Required	Main Reagents	Toxic Compound	Cost Per Sample (US$)
Chelex-100 method	≈25 min	Chelex-100, Proteinase K	None	< 0.2
Commercial kit	> 3 h	TL Buffer, OB Protease, BL Buffer, HB Buffer, DNA Wash Buffer, Elution Buffer, HiBind DNA Column	Isopropanol	≈2

### 3.3. Comparison of DNA Extraction Methods by PCR, Sequencing and qPCR

**Fig 2:**
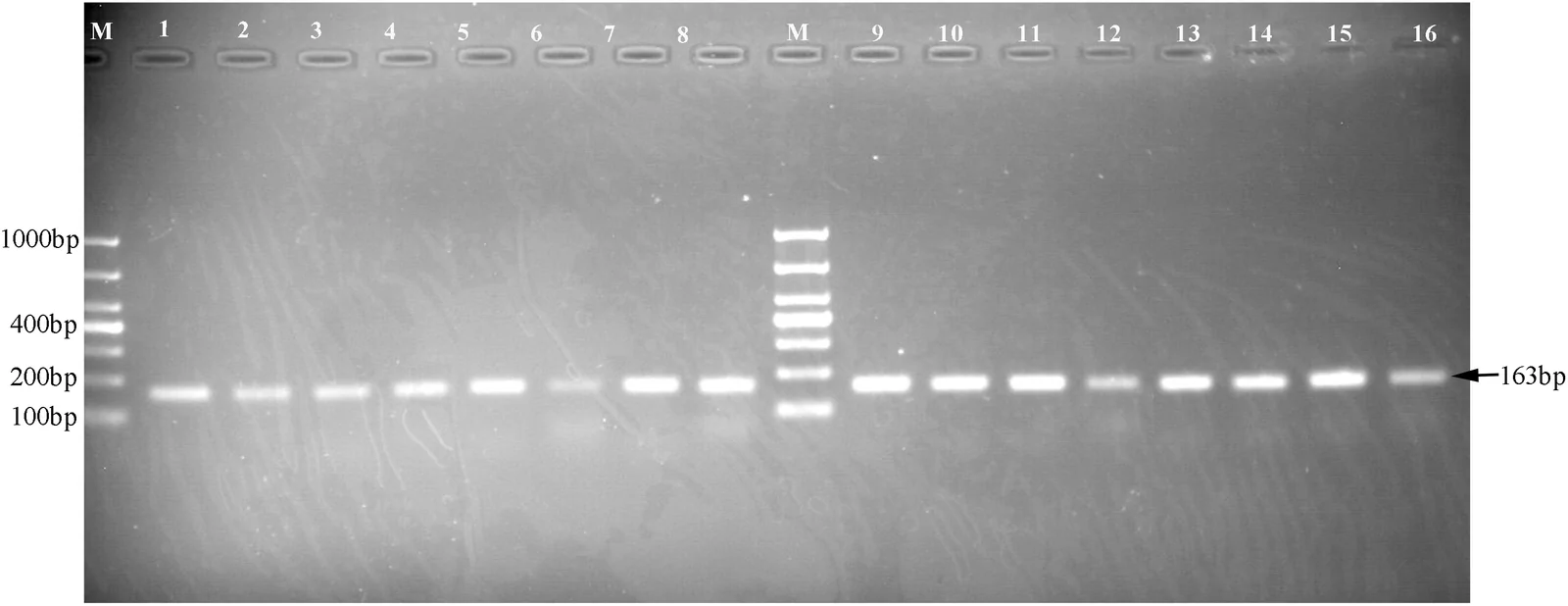
PCR amplification of the TLR9 gene fragment in various adult zebrafish tissues. Lanes 1,3,5,7,9,11,13 and 15: gDNA extracted from zebrafish muscle, liver, intestine, eggs, brain, spleen, heart, and kidney tissues, respectively, by using a commercial kit method; Lanes 2,4,6,8,10,12,14 and 16: gDNA extracted from the same set of zebrafish tissues in turn by using the Chelex-100 resin method; M: DL1000 DNA marker (3591Q, Takara, Japan).

**Fig 3:**
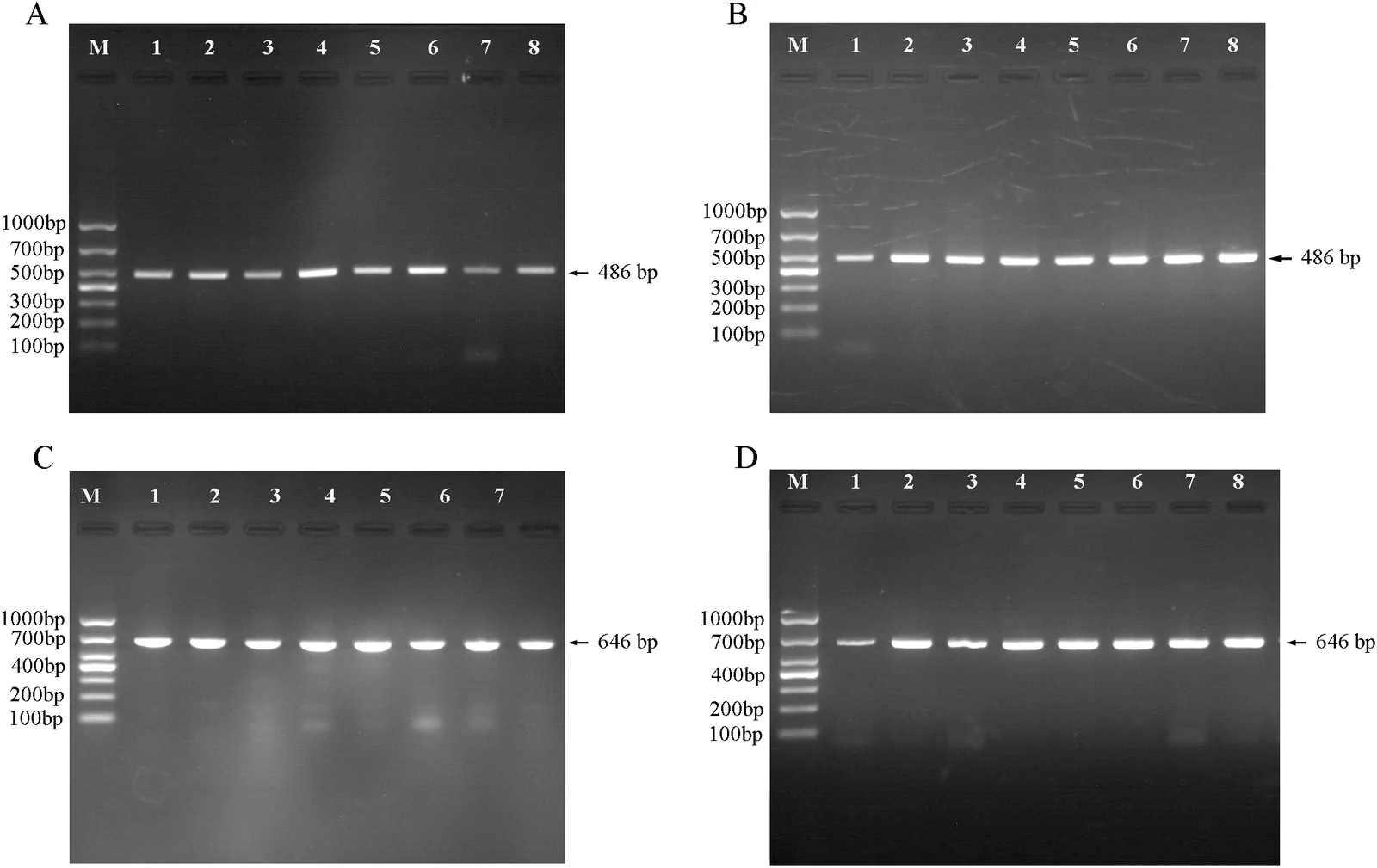
PCR amplification of β-actin and 18S rRNA fragments in various adult zebrafish tissues using 2% agarose gel electrophoresis. (A) PCR products of β-actin from extracted gDNA by the Chelex-100 resin method; (B) PCR products of β-actin from extracted gDNA by a commercial kit method; (C) PCR products of 18S rRNA from extracted gDNA by the Chelex-100 resin method; (D) PCR products of 18S rRNA from extracted gDNA by a commercial kit method. Lanes 1,2,3,4,5,6,7 and 8: The templates are gDNA extracted from zebrafish intestine, muscle, eggs, brain, liver, spleen, heart and kidney tissues in turn; M: DL1000 DNA marker.

**Fig 4:**
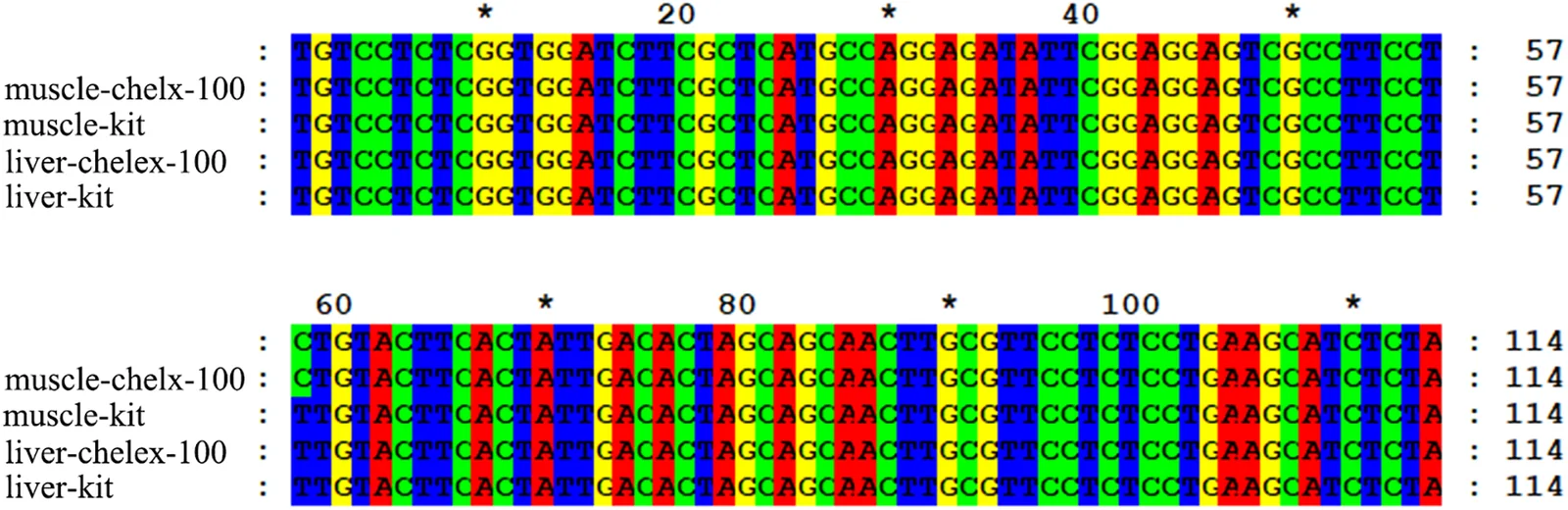
Alignment of PCR-amplified TLR9 gene fragments from zebrafish liver and muscle tissues following DNA extraction using the Chelex-100 resin method or a commercial kit.

**Fig 5:**
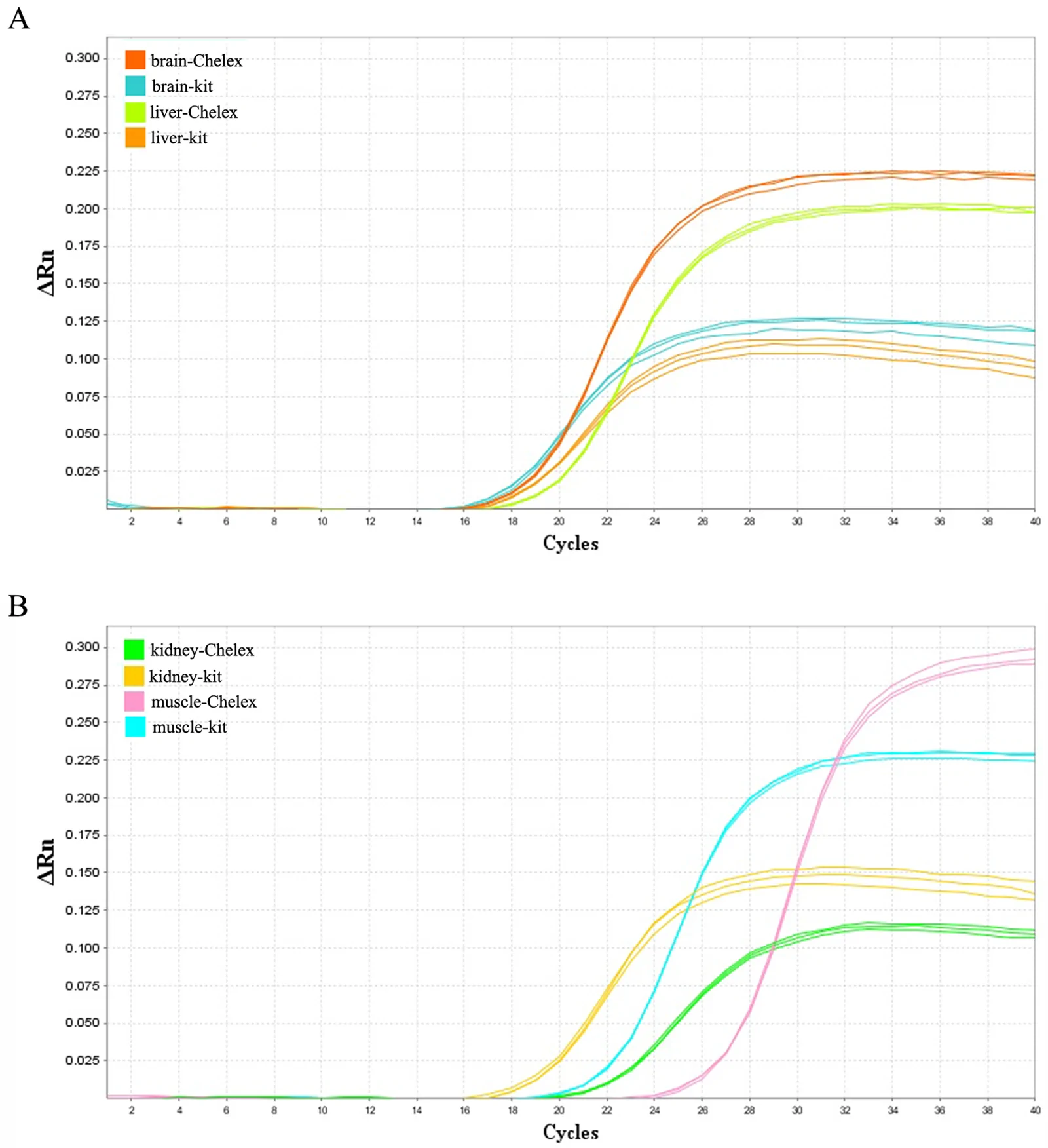
qPCR amplification curves of TLR9 in adult zebrafish brain and liver (A), as well as kidney and muscle (B), amplified using gDNA extracted by either the Chelex-100 resin method or a commercial kit.

**Fig 6:**
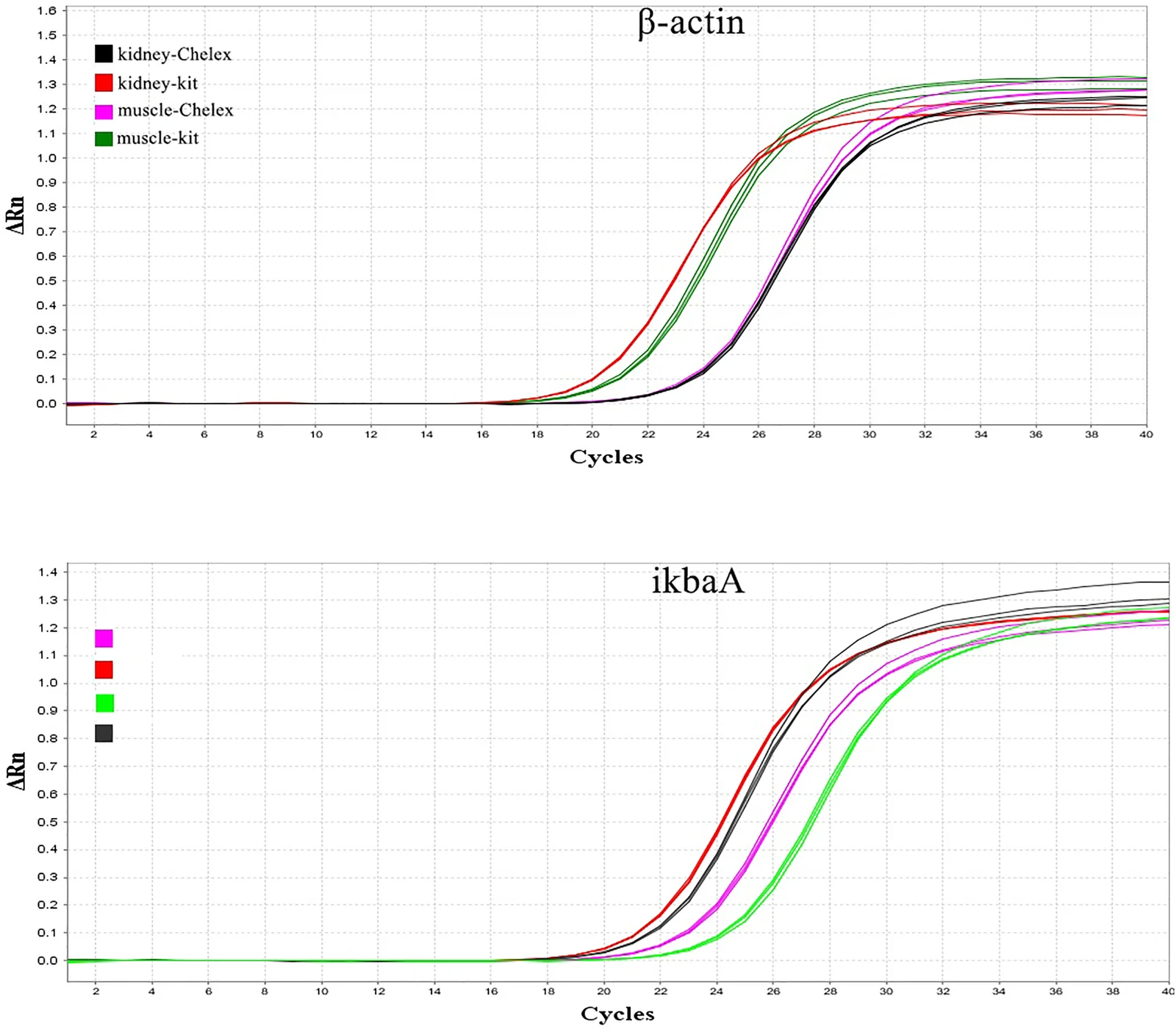
qPCR amplification curves of β-actin and ikbaA using gDNA extracted from adult zebrafish tissues as the templates by either the Chelex-100 resin method or a commercial kit.

PCR amplification was performed to evaluate the quality of the extracted gDNA using both the Chelex-100 resin method and a commercial kit. The fragments of *β-actin*, *18S rRNA*, and *TLR9* were successfully amplified in all samples, with consistent amplification profiles observed between the two methods via 2% agarose gel electrophoresis (Figs 2 and 3). The PCR products of the TLR9 fragments from liver and muscle tissues were subsequently subjected to forward sequencing, and the resulting sequences were aligned against the reference sequence (accession number: NC_133183.1) retrieved from the database of the National Center for Biotechnology Information. Sequence alignment revealed high consistency among the fragments derived from gDNA extracted by both methods and the reference sequence (Fig 4), indicating that the gDNA obtained via the Chelex-100 resin method can be directly used for PCR-based sequencing and yields accurate sequence data. To further evaluate extraction efficiency, qPCR was conducted on *TLR9*, *β-actin*, and *ikbaA* using gDNA obtained from adult zebrafish tissues via both methods as the template. The amplification curves and Ct values are shown in Figs 5, 6, and Table 3, respectively. Both methods yielded good results, but compared with the commercial kit method, the Chelex-100 resin method resulted in higher Ct values across all tissues for all three genes (β-actin, TLR9, and ikbaA). These findings suggest that the quality of gDNA extracted via the Chelex-100 resin method is sufficient for use in qPCR assay. However, the low purity of gDNA from the Chelex-100 resin method may affect the efficiency of PCR amplification and the binding of fluorescent dyes to DNA molecules.

**Table 3:** Ct Values of qPCR using gDNA extracted from adult zebrafish tissues as the templates with two different methods.

Tissue	β-actin		TLR9		ikbaA	
	Chelex	kit	Chelex	kit	Chelex	kit
heart	22.09 ± 0.14	19.90 ± 0.15	21.03 ± 0.08	19.86 ± 0.08	24.17 ± 0.08	22.24 ± 0.09
liver	22.72 ± 0.12	20.30 ± 0.16	19.79 ± 0.04	18.95 ± 0.02	25.10 ± 0.14	22.50 ± 0.06
spleen	21.09 ± 0.05	19.09 ± 0.13	25.68 ± 0.09	21.34 ± 0.09	23.33 ± 0.07	21.39 ± 0.09
kidney	23.36 ± 0.07	19.83 ± 0.04	22.73 ± 0.09	19.32 ± 0.15	25.62 ± 0.12	22.07 ± 0.06
intestine	22.31 ± 0.06	22.31 ± 0.06	26.61 ± 0.11	22.95 ± 0.13	25.81 ± 0.14	24.58 ± 0.07
brain	21.30 ± 0.09	19.59 ± 0.11	18.56 ± 0.04	18.13 ± 0.11	23.73 ± 0.09	22.01 ± 0.03
muscle	23.29 ± 0.08	20.67 ± 0.11	26.23 ± 0.05	21.76 ± 0.04	25.75 ± 0.11	23.08 ± 0.09
eggs	24.17 ± 0.18	22.58 ± 0.09	25.16 ± 0.10	19.08 ± 0.02	26.83 ± 0.11	25.46 ± 0.36

## 4. Discussion

The zebrafish is a widely used vertebrate model in studies of development, physiology, behavior, toxicology, and disease [36–39]. Efficient gDNA extraction from adult zebrafish tissues is essential for molecular applications such as genotyping, gene expression analysis, and pathogen detection. In this study, we developed a rapid, cost-effective, and reliable method for gDNA extraction from adult zebrafish tissues using Chelex-100 resin combined with proteinase K, a well-documented alternative to traditional phenol‒chloroform extraction or commercial kits, with advantages in simplicity, speed, cost, and the avoidance of hazardous reagents.

Optimization experiments were conducted by evaluating the concentration and purity of gDNA extracted from zebrafish muscle tissue following incubation at 56 °C for varying durations (5, 15, 30, and 60 min) and subsequent boiling at 100 °C for different durations (5, 8, and 10 min). The highest DNA concentration was obtained following incubation at 56 °C for 15 min and 100 °C for 10 min. This finding aligns with previous reports that the alkalinity of Chelex-100 suspensions, combined with thermal denaturation, promotes cell membrane disruption and nucleic acid release [22]. The addition of proteinase K prior to heating likely increases tissue digestion and chromatin decondensation, facilitating more efficient DNA release and reducing extraction time, which is particularly beneficial for fibrous or protein-rich tissues.

We then systematically compared this optimized Chelex-100 method with a commercial kit across several adult zebrafish tissues (muscle, liver, heart, eggs, spleen, kidney, intestine, and brain) by measuring the concentration and purity of extracted gDNA. In most tissues, the DNA concentration obtained using the Chelex-100 resin method was greater than that obtained using the commercial kit. This may be due to reduced sample loss during the procedure as well as to a higher level of coextracted impurities. Notably, its purity was consistently lower (A260/A280 ratios = 1.36–1.83) than that of the kit method (A260/A280 ratios = 1.84–2.02) because the protocol omits purification steps to remove impurities, including proteins or other coextracted materials, which represents a substantial difference and may affect applications that require cleaner templates, such as next-generation sequencing library preparation.

For downstream validation, conventional PCR of *β-actin*, 1*8S rRNA*, and *TLR9* and sequencing of the TLR9 gene fragment were performed to confirm DNA integrity and specificity. These results showed that Chelex-100-extracted gDNA was sufficient for amplification of the target fragments under the conditions tested, and the sequencing results of TLR9 were consistent with those obtained from kit-extracted gDNA, suggesting that the presence of certain amounts of impurities, such as RNA and proteins, in the DNA sample does not significantly affect the PCR amplification results. qPCR analysis demonstrated that both methods produced favorable amplification curves. However, compared with the commercial kit, the Chelex-100 resin method yielded systematically higher Ct values in several tissues (Table 3), indicating that the lower purity of the template affects the sensitivity of the qPCR detection results, especially for some samples near the detection limit. Therefore, while the Chelex-100 method is suitable for routine PCR-based applications, researchers should exercise caution when applying it to quantitative assays requiring high sensitivity and precision.

Overall, the optimized Chelex-100 resin method provides a rapid and low-cost option for extracting gDNA from adult zebrafish tissues (Table 2). The results of the present study support the use of this method as a practical alternative for routine PCR, sequencing, and qPCR. Given the expanding use of zebrafish in infectious disease and immunological research [37,40,41], this method could facilitate rapid screening for common pathogens such as Pseudoloma neurophilia or Edwardsiella ictaluri in both aquaculture and research. Notably, the impact of consistently lower DNA purity needs to be specifically evaluated when other tissues and pathogens are involved.

## 5. Conclusions

In the present study, we established a Chelex-100 resin-based method for gDNA extraction from various adult zebrafish tissues, with a total processing time of approximately 25 min, yielding higher concentrations but lower purity due to omitting purification steps to remove impurities. Compared with a commercial kit, the Chelex-100 resin method delivered comparable performance in conventional PCR amplification of target fragments (*β*-*actin*, *18S rRNA*, and *TLR9*), Sanger sequencing of TLR9 fragments, and qPCR, supporting its utility for routine PCR and basic molecular assays. In addition, it offers the advantages of low cost (<$0.20 per sample), rapidity (~25 min), and the avoidance of hazardous reagents compared with the commercial kit method (approximately $2.00 per sample, > 3 h). However, the Ct values in the qPCR results across all the tested tissues were higher because of its lower purity, suggesting a reduced sensitivity that may affect the quantification of low-abundance targets or samples near the detection limit. In summary, this Chelex-100/proteinase K protocol offers a rapid, economical, and practical alternative for adult zebrafish gDNA extraction, particularly for routine PCR-based applications, but its limitations in terms of purity and qPCR sensitivity should be carefully considered and addressed depending on the specific experimental requirements.

## Supporting information

S1 Table Primers used for PCR and qPCR. (DOCX)

S2 Table Data used to calculate the averages presented on Fig 1. (DOCX)

S1 Fig Original agarose gel electrophoresis images. Raw gel images showing PCR amplification of TLR9, β-actin, and 18S rRNA gene fragments in various tissues of adult zebrafish.
(PDF)
