# Supplementary material for: A rapid and cost-effective Chelex-100 resin-based method for genomic DNA extraction from adult zebrafish tissues
Source: PLoS One. 2026 Sep 18;21(9):e0358591. doi: 10.1371/journal.pone.0358591 (PMC13588526; doi:10.1371/journal.pone.0358591)
Supplement: Supplementary file 1 [file pone.0358591.s003.pdf]

origianl image

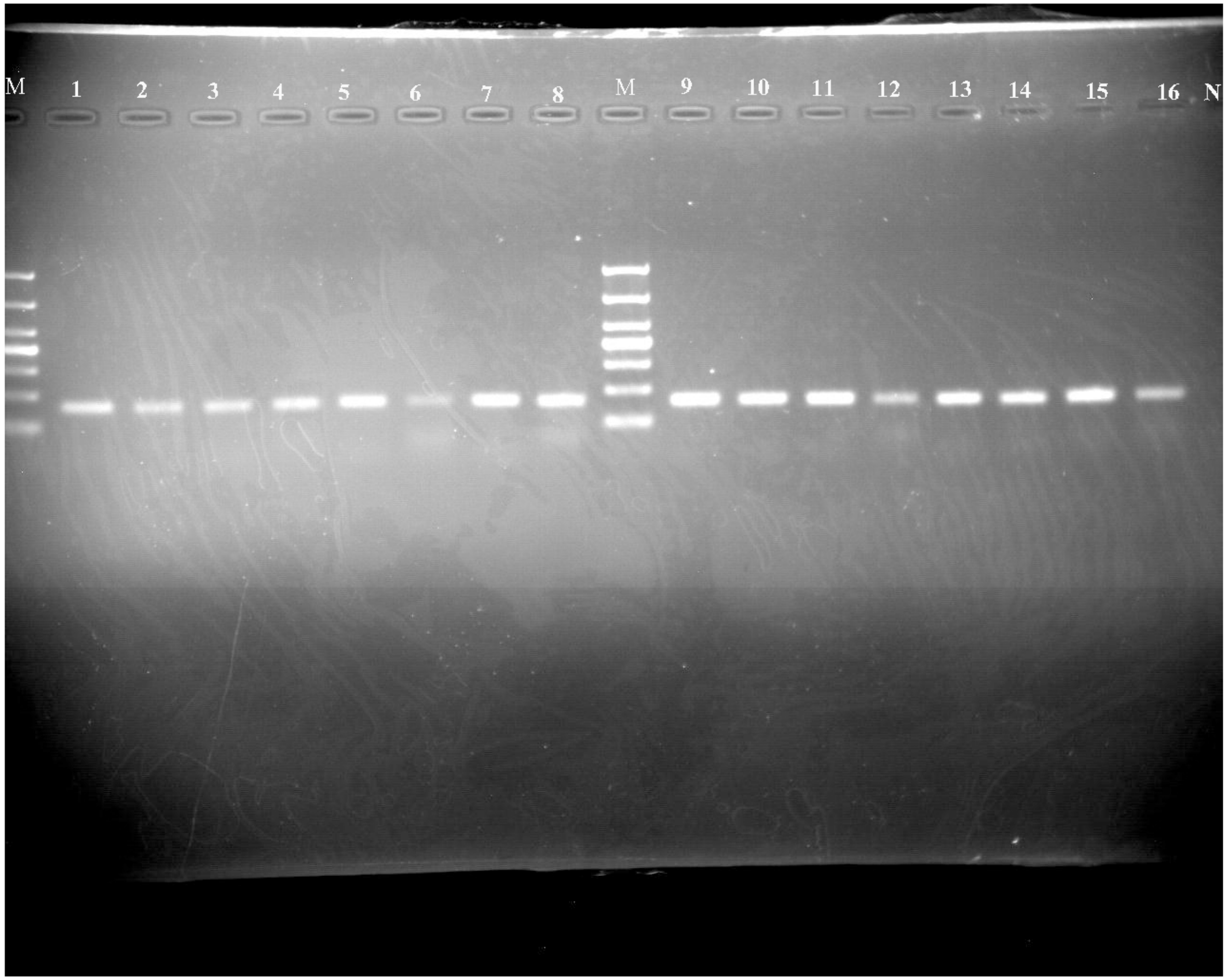

The raw image of PCR amplification of the TLR9 gene fragment in various adult zebrafish tissues. M: DL1000 DNA Marker (3591Q, Takara, Japan); lanes 1,3,5,7,9,11,13,15: templates are gDNA from zebrafish muscle, liver, intestine, eggs, brain, spleen, heart, and kidney tissues in turn, extracted using a commercial kit; lanes 2,4,6,8,10,12,14,16: templates are gDNA from the same set of zebrafish tissues in turn, extracted using the

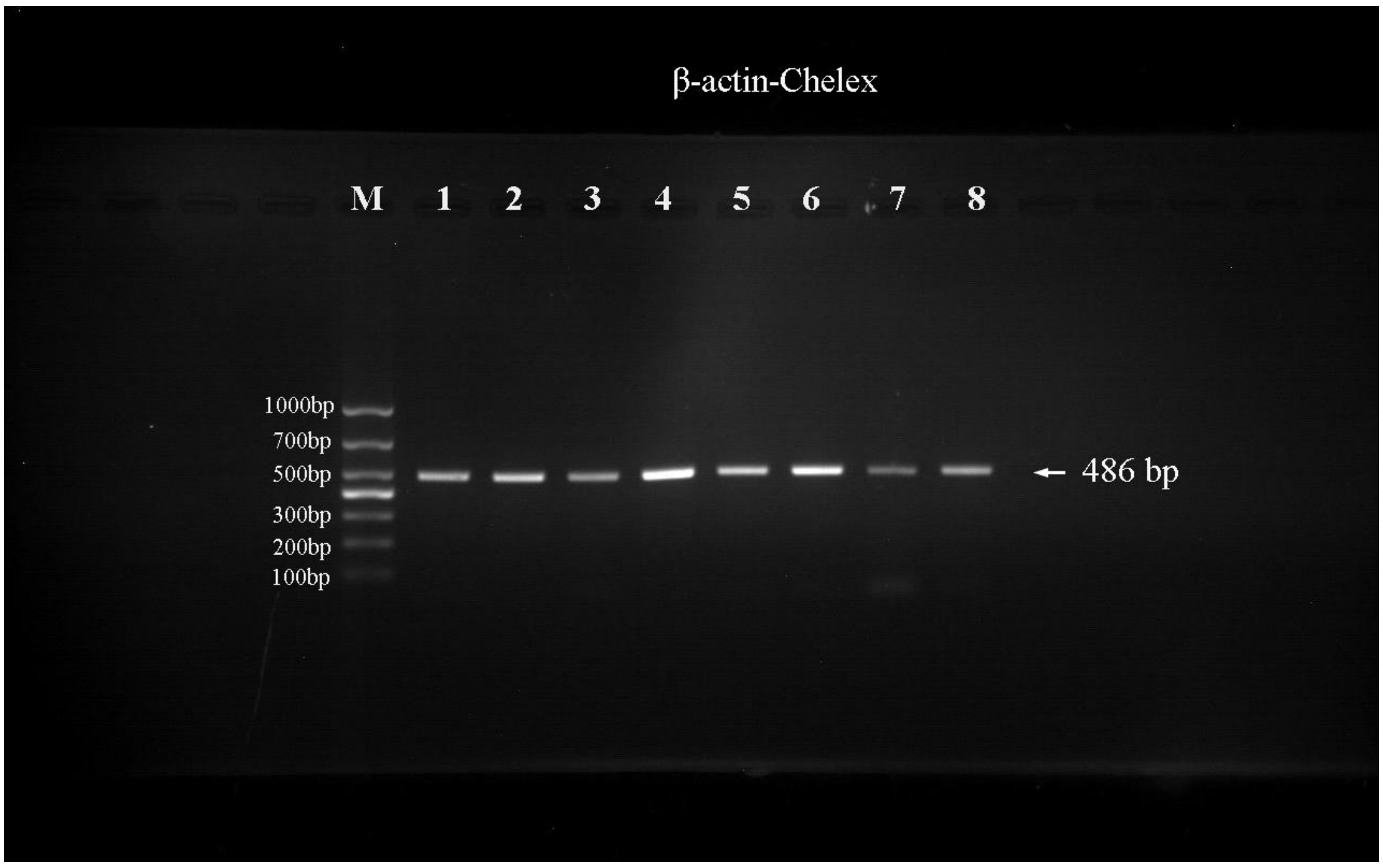

The raw image of PCR amplification of β-actin fragments in various adult zebrafish tissues using the extracted gDNA by the Chelex-100 resin method as the templates. Lanes 1,2,3,4,5,6,7 and 8: The templates are gDNA extracted from zebrafish intestine, muscle, egg, brain, liver, spleen, heart and kidney tissues in turn; M: DL1000 DNA marker.

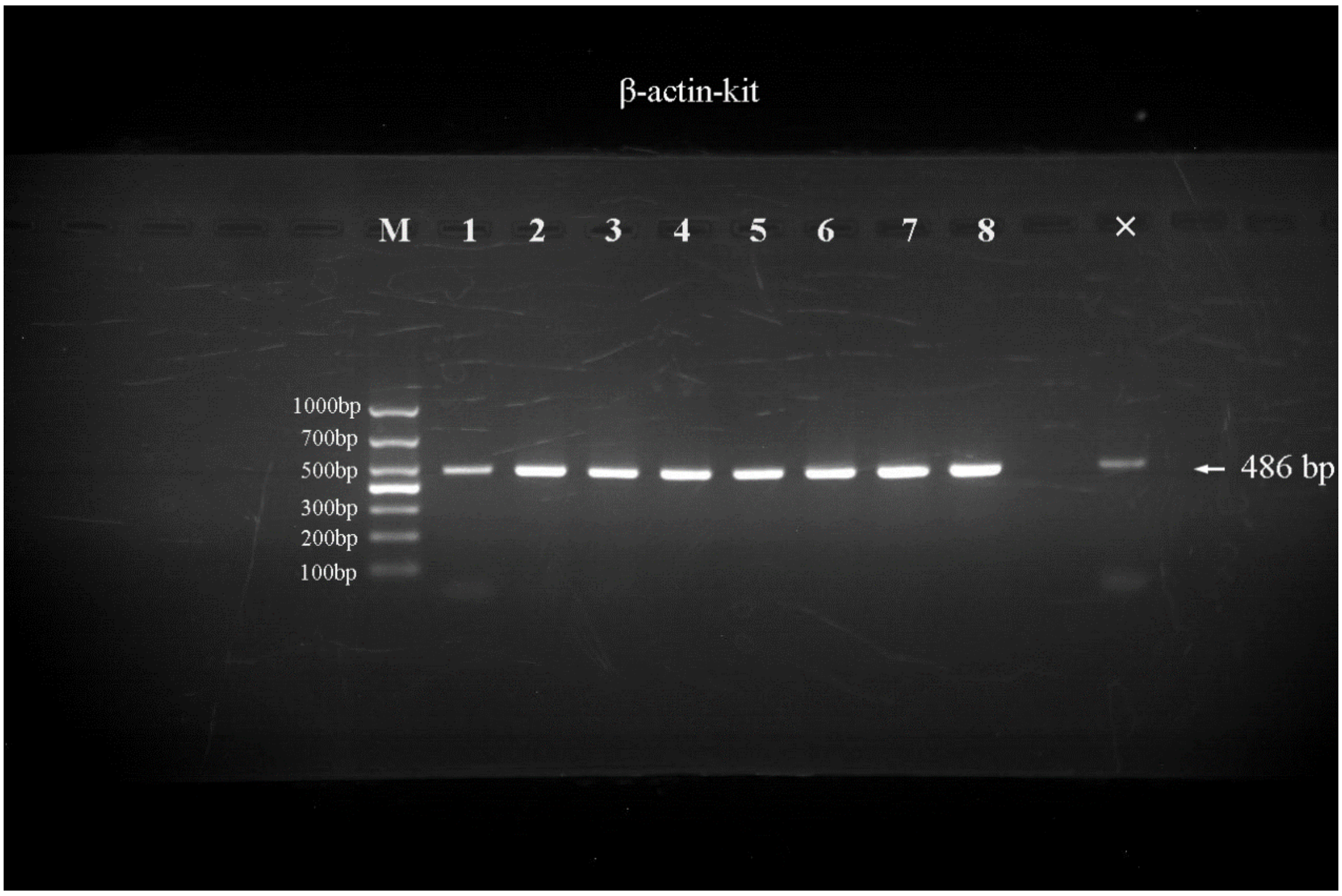

The raw image of PCR amplification of β-actin fragments in various adult zebrafish tissues using the extracted gDNA by a commercial kit method as the templates. Lanes 1,2,3,4,5,6,7 and 8: The templates are gDNA extracted from zebrafish intestine, muscle, egg, brain, liver, spleen, heart and kidney tissues in turn; M: DL1000 DNA marker.

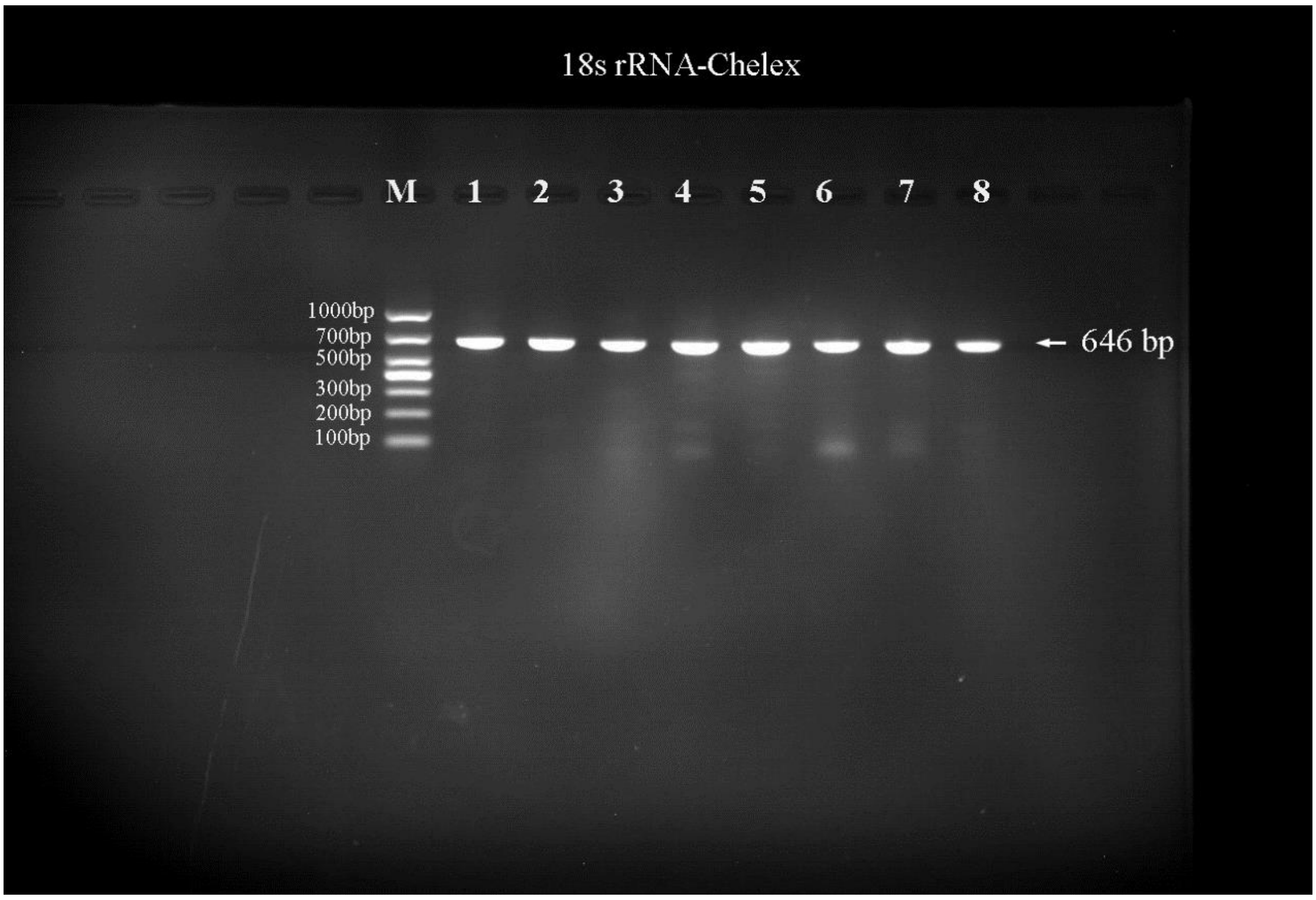

The raw image of PCR amplification of 18s rRNA fragments in various adult zebrafish tissues using the extracted gDNA by the Chelex-100 resin method as the templates. Lanes 1,2,3,4,5,6,7 and 8: The templates are gDNA extracted from zebrafish intestine, muscle, egg, brain, liver, spleen, heart and kidney tissues in turn; M: DL1000 DNA marker.

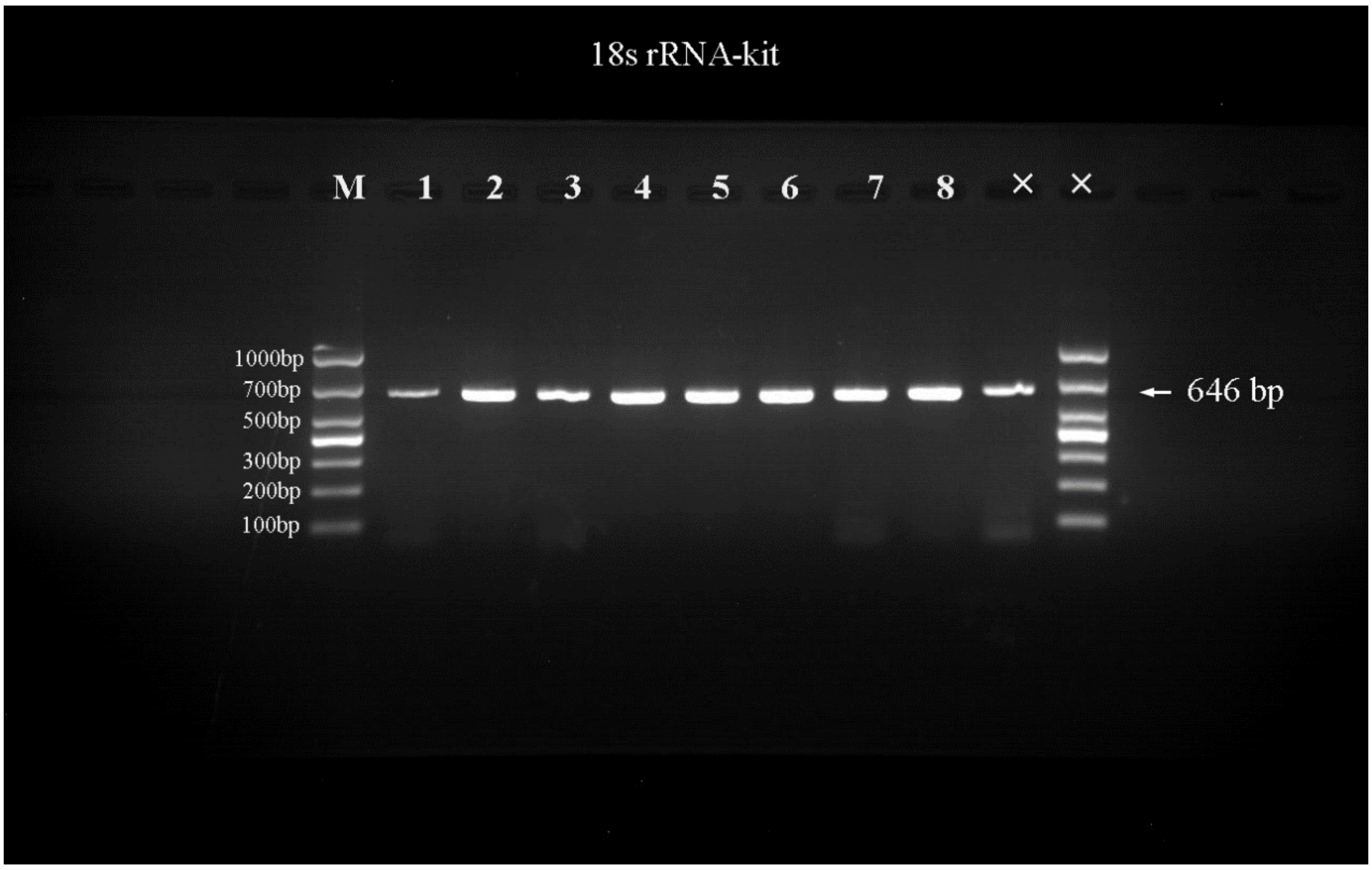

The raw image of PCR amplification of 18s rRNA fragments in various adult zebrafish tissues using the extracted gDNA by a commercial kit method as the templates. Lanes 1,2,3,4,5,6,7 and 8: The templates are gDNA extracted from zebrafish intestine, muscle, egg, brain, liver, spleen, heart and kidney tissues in turn; M: DL1000 DNA marker.
