# Supplementary material for: A rapid and cost-effective Chelex-100 resin-based method for genomic DNA extraction from adult zebrafish tissues
Source: PLoS One. 2026 Sep 18;21(9):e0358591. doi: 10.1371/journal.pone.0358591 (PMC13588526; doi:10.1371/journal.pone.0358591)
Supplement: Supplementary file 2 [file pone.0358591.s001.docx]

S1 Table. Primers used for PCR and qPCR

| Primer Name | Sequence (5’→ 3’ ) | Annealing temperature (℃) | | Amplicon size (bp) | Use |
| --- | --- | --- | --- | --- | --- |
| β-actin-F | TCAGCAAGCAGGAGTACGAT | | 60 | 486 | PCR |
| β-actin-R | GGGACATCCCTTATTCACAT | |  |  | PCR |
| 18S rRNA-F | GCCGACTGCGCCCTTATGAA | | 70 | 646 | PCR |
| 18S rRNA-R | GGCTGAACGCCACTTGTCCC | |  |  | PCR |
| β-actin-QF | AGGTCATCACCATCGGCAAT | | 58 | 217 | qPCR |
| β-actin-QR | GATGTCCACGTCGCACTTCA | |  |  | qPCR |
| ikbaA-QF | GGTGGAAAGACTCCTGAAAGC | | 58 | 164 | qPCR |
| ikbaA-QR | TGTAGTTAGGGAAGGTGAGAATG | |  |  | qPCR |
| TLR9-QF | TCACTACACAGGTTCACT | | 58 | 163 | PCR and qPCR |
| TLR9-QR | TAGAGATGCTTCAGGAGAG | |  |  | PCR and qPCR |
