# Supplementary material for: A rapid and cost-effective Chelex-100 resin-based method for genomic DNA extraction from adult zebrafish tissues
Source: PLoS One. 2026 Sep 18;21(9):e0358591. doi: 10.1371/journal.pone.0358591 (PMC13588526; doi:10.1371/journal.pone.0358591)
Supplement: Supplementary file 3 [file pone.0358591.s002.docx]

S2 Table. Data used to calculate the averages presented on Fig 1.

Data used to calculate the averages presented on Fig 1A.

| Incubation time(min) | DNA concentration (ng/µl) | A260/A280 |
| --- | --- | --- |
| 5 | 168.25 | 1.339 |
|  | 155.15 | 1.313 |
|  | 14.9 | 1.343 |
| 15 | 186.35 | 1.289 |
|  | 185.6 | 1.267 |
|  | 187.9 | 1.284 |
| 30 | 183.25 | 1.276 |
|  | 178.9 | 1.298 |
|  | 195.85 | 1.297 |
| 60 | 150.5 | 1.293 |
|  | 143.2 | 1.272 |
|  | 155.3 | 1.275 |

Data used to calculate the averages presented on Fig 1B.

| Boiling time(min) | DNA concentration (ng/µl) | A260/A280 |
| --- | --- | --- |
| 5 | 164.09 | 1.322 |
|  | 158.09 | 1.321 |
|  | 161.09 | 1.314 |
| 8 | 182.66 | 1.349 |
|  | 185.56 | 1.352 |
|  | 184.11 | 1.326 |
| 10 | 196.16 | 1.369 |
|  | 188.73 | 1.372 |
|  | 192.44 | 1.381 |
